# Simulation of O_3_ and NO_*x*_ in São Paulo street urban canyons with VEIN (v0.2.2) and MUNICH (v1.0)

**DOI:** 10.5194/gmd-14-3251-2021

**Published:** 2021-06-03

**Authors:** Mario Eduardo Gavidia-Calderón, Sergio Ibarra-Espinosa, Youngseob Kim, Yang Zhang, Maria de Fatima Andrade

**Affiliations:** 1 Departamento de Ciências Atmosféricas, Instituto de Astronomia, Geofísica e Ciências Atmosféricas, Universidade de São Paulo, 05508-090, São Paulo, Brazil; 2 CEREA, Joint Laboratory École des Ponts ParisTech/EDF R&D, Université Paris-Est, 77455 Champs-sur-Marne, France; 3 Department of Civil and Environmental Engineering, Northeastern University, Boston, MA 02115, USA

## Abstract

We evaluate the performance of the Model of Urban Network of Intersecting Canyons and Highways (MUNICH) in simulating ozone (O_3_) and nitrogen oxides (NO_*x*_) concentrations within the urban street canyons in the São Paulo metropolitan area (SPMA). The MUNICH simulations are performed inside the Pinheiros neighborhood (a residential area) and Paulista Avenue (an economic hub), which are representative urban canyons in the SPMA. Both zones have air quality stations maintained by the São Paulo Environmental Agency (CETESB), providing data (both pollutant concentrations and meteorological) for model evaluation. Meteorological inputs for MUNICH are produced by a simulation with the Weather Research and Forecasting model (WRF) over triple-nested domains with the innermost domain centered over the SPMA at a spatial grid resolution of 1 km. Street coordinates and emission flux rates are retrieved from the Vehicular Emission Inventory (VEIN) emission model, representing the real fleet of the region. The VEIN model has an advantage to spatially represent emissions and present compatibility with MUNICH. Building height is estimated from the World Urban Database and Access Portal Tools (WUDAPT) local climate zone map for SPMA. Background concentrations are obtained from the Ibirapuera air quality station located in an urban park. Finally, volatile organic compound (VOC) speciation is approximated using information from the São Paulo air quality forecast emission file and non-methane hydrocarbon concentration measurements. Results show an overprediction of O_3_ concentrations in both study cases. NO_*x*_ concentrations are underpredicted in Pinheiros but are better simulated in Paulista Avenue. Compared to O_3_, NO_2_ is better simulated in both urban zones. The O_3_ prediction is highly dependent on the background concentration, which is the main cause for the model O_3_ overprediction. The MUNICH simulations satisfy the performance criteria when emissions are calibrated. The results show the great potential of MUNICH to represent the concentrations of pollutants emitted by the fleet close to the streets. The street-scale air pollutant predictions make it possible in the future to evaluate the impacts on public health due to human exposure to primary exhaust gas pollutants emitted by the vehicles.

## 1 Introduction

Street urban canyons are structures formed by a street and its flanked buildings (Oke et al., 2017). Due to their proximity to emissions from vehicles and their side function as a compartment that limits pollutant dispersion, the street and the associated urban canyons are considered pollutant hotspots (Zhong et al., 2016). As more people start to live in urban areas (United Nations, 2018) and the ubiquity of urban canyons in cities grows, pedestrians, commuters, bikers, and drivers are being exposed to high pollutant concentrations every day (Vardoulakis et al., 2003). Consequently, the study of air pollution inside urban canyons is an important matter when dealing with studies of human health exposure related to traffic emissions.

To estimate the real impact of the pollutants on human health, it is necessary to obtain accurate pollutant concentrations and the lengths of exposure. Most cities are not covered by a high-density network of air quality stations. Even though the measurements provide precise information, it is expensive and also very difficult to cover all of the impacted areas of a city (Zhong et al., 2016). One alternative, that is starting to be contemplated, is the use of numerical modeling to represent the pollutant behavior in urban canyons, which has the advantage of producing pollutant concentration information at high temporal and spatial resolutions.

Computational fluid dynamics (CFD) models are considered to be the best modeling approach to understand air pollutant dispersion inside the urban areas. Due to the limitations of high computational resources, these models cannot be applied for long-time simulation periods nor for a large area (Fellini et al., 2019; Thouron et al., 2019).

A new type of model, the urban-/local-scale operational model, overcomes these limitations by applying simplifications on urban geometry and parameterizations of the mass transfer processes of air pollutants inside the urban canyons. The Operational Street Pollution Model (OSPM) and the Atmospheric Dispersion Model System (ADMS-urban) are two of the most popular operational models which have already been tested for different cities around the world (Berkowicz et al., 1997; McHugh et al., 1997). Their main advantage is that they calculate pollutant concentrations when sources and receptors are in the same street urban canyon, but they present a limited treatment for the pollutant transfer between streets and intersections (Carpentieri et al., 2012).

Street-network models are also operational, having the advantage of dealing with the transport of pollutants in city street intersections. The SIRANE model uses parametric relations to solve advection on the streets, the dispersion in the street intersections, and interchange between the streets and the over-roof atmosphere (Soulhac et al., 2011, 2012). Background concentrations at the over-roof atmosphere are estimated using a Gaussian plume model. This estimation method inhibits a comprehensive atmospheric chemistry treatment.

Recently, the Model of Urban Network of intersecting Canyons and Highways (MUNICH) was developed by Kim et al. (2018a) using a similar parameterization to that of SIRANE. MUNICH includes improvements in the treatment of the mean wind profile inside the urban canyon and the turbulent vertical mass transfer at the top of the street. It solves pollutant reactions using a chemical mechanism, so it can also simulate the production of ozone inside the urban canyons. MUNICH has been used to simulate ozone (O_3_) and nitrogen oxides (NO_*x*_) by Wu et al. (2020) in the Tianhe district of Guangzhou city and NO_*x*_ as part of the Street in Grid (SinG) model in Kim et al. (2018a), Thouron et al. (2019), and Lugon et al. (2020) in the Paris region.

Significant information is required to run this kind of model. It is explained by Vardoulakis et al. (2003) that, in general, these models need at least information from traffic data, emissions, meteorological data, street geometry, and background concentrations. Recently, the Vehicular Emission Inventory (VEIN) model was developed by Ibarra-Espinosa et al. (2018) using information for São Paulo. VEIN is suitable for use in street-network models because it uses the traffic flow, emission factors, and street morphology (i.e., intersection coordinates) to calculate the vehicular emissions. As a matter of fact, due to its architecture, it can be used together with MUNICH.

In Brazil, previous studies of air quality in urban canyons dealt with measurements of black carbon and O_3_ inside a street canyon in Londrina’s city center (Krecl et al., 2016), and dispersion of NO_*x*_ was simulated in Curitiba with the ENVI-met model (Krüger et al., 2011). To our knowledge, this is the first study of modeling O_3_ and NO_*x*_ inside street urban canyons in the São Paulo metropolitan area (SPMA), the biggest megacity in South America, where it is very often the exceedance of O_3_ state air quality standard (Andrade et al., 2017).

As the management of secondary pollutants remains a challenge in SPMA, we aim to evaluate MUNICH operational street-network model to simulate O_3_ and NO_*x*_ concentration inside urban canyons, coupled with the VEIN emission model, to build a street-level air quality modeling system. This modeling system can be used in air quality and traffic management of the São Paulo neighborhoods, in studies of health effects from traffic emission exposure, in future urban planning, and post-accident analysis.

## 2 Data and methods

The experiment consisted of carrying out simulations of O_3_, NO_*x*_, NO, and NO_2_ concentrations inside the SPMA urban street canyons with the MUNICH model. To evaluate model performance, the model results are compared against the measurements from the São Paulo Environmental Agency (CETESB) air quality network. We choose the Pinheiros urban area to test the model, where there is an air quality station in a mixed residential–commercial area. Once MUNICH and VEIN are calibrated, a study case is prepared by calculating the pollutant concentration inside Paulista Avenue, the economic central area of the city with high canyons. The selected study period covers the week from 6 to 13 October of 2014. This period is chosen before dry weather conditions in SPMA, a period of high O_3_ concentrations (Carvalho et al., 2015), the availability of data, and the availability of the emission inventory developed for a typical week in October 2014 (Ibarra-Espinosa et al., 2020).

### 2.1 MUNICH model

MUNICH is conceptually based on the SIRANE model (Soulhac et al., 2011). It has two main components: the street-canyon component, which deals with and solves pollutant concentrations inside the urban-canopy volume, and the intersection component, which calculates the pollutant concentrations inside the intersection volume. MUNICH differs from SIRANE in the treatment of the vertical flux by turbulent diffusion at the roof level (Schulte parameterization; Schulte et al., 2015) and in the mean wind velocity within the street canyon (Lemonsu parameterization; Lemonsu et al., 2004). Currently, MUNICH solves gas-phase pollutants based on the Carbon Bond mechanism version 5 (CB05). Further information is detailed in Kim et al. (2018a).

### 2.2 VEIN emission model

VEIN is an R package (R Core Team, 2020) to estimate vehicular emissions at the street level. VEIN imports functions from the Spatial Features package (Pebesma, 2018), which represent different types of geometries in space and perform geoprocessing tasks, from the data table package (Dowle and Srinivasan, 2019) to perform fast aggregation of databases, and from the units package (Pebesma et al., 2016) to provide binding to the udunits library (https://www.unidata.ucar.edu/software/udunits/, last access: 28 May 2021). VEIN includes a function to process vehicular flow at each street to generate activity traffic data, different emissions factors, and different sets of emissions calculation and post-processing tools (Ibarra-Espinosa et al., 2018). Specifically, the emissions factors are based on emissions certification tests with dynamometer measurements in laboratories (CETESB, 2015).

### 2.3 MUNICH input data

Urban canyon models required detailed input information, such as building height and street geometry. Their performance depends on the quality of this information (Vardoulakis et al., 2003). In recent years, new tools have been developed to generate this information. Table 1 summarizes the model input used in this simulation experiment.

#### 2.3.1 Emissions and street links coordinates

The vehicular fleet is the principal source of air pollution in SPMA (Andrade et al., 2015, 2017). The particularity of this fleet is the extensive use of biofuels (i.e., gasohol, ethanol, and biodiesel). During 2014, vehicular emissions were responsible for emitting 97 % of CO, 82 % of volatile organic compounds (VOCs), 78 % of NO_*x*_, and 40 % of particulate matter (CETESB, 2015). Vehicular emissions inside SPMA streets were estimated using the VEIN emission model (Ibarra-Espinosa et al., 2018).

Street links are segments of roads split at each vertex. Then, a road can be composed of many links. Emission rates inside these street links in the VEIN model are calculated using 104 million GPS vehicles coordinates in southeast Brazil (Ibarra-Espinosa et al., 2019). The GPS dataset is assigned to the OpenStreetMap (2017) dataset and once traffic flow is obtained, the vehicular compositions are generated and assigned to each emission factor reported by CETESB (2015). Emission factors are transformed into speed functions, and then the average speed calculated at each street is used to obtain more representative emissions at each hour of a week. In addition, the estimation was calibrated with fuel consumption for the year 2014. Ibarra-Espinosa et al. (2020a) described all details regarding the emission estimation with the emissions dataset in g h^−1^ available at https://github.com/ibarraespinosa/ae1 (last access: 28 May 2021).

The emissions dataset presents two aspects that need to be discussed. The first one is that there are some differences between the traffic flow from travel demand model (TDM) outputs and GPS (Ibarra-Espinosa et al., 2019, 2020a). The ratio between traffic flows from TDM and GPS for our study is 2.22. Regarding the emissions factors used to estimate the emissions, they are based on average measurement of emissions certification tests (CETESB, 2015); therefore, they may underestimate real-drive emissions (Ropkins et al., 2009). For instance, the real-world emission factors derived from tunnel measurements in São Paulo for NO_*x*_ were 0.3 g km^−1^ for light vehicles and 9.2 g km^−1^ for heavy vehicles (Pérez-Martínez et al., 2014), while the respective fleet-weighted CETESB (2015) emission factors are 0.26 and 6.68 g km^−1^, as shown in Fig. S1 in the Supplement, resulting in ratios of 1.11 and 1.38. Then, if we consider the mean emission-factor ratio *(*1.11+1.38*)/*2 multiplied by the mentioned traffic flow ratio (2.22) results, the NO_*x*_ emissions might be approximately 2.73 higher than those estimated using pure CETESB (2015) data. Consequently, we expect that air quality simulations for NO_*x*_ might be lower than observations.

**Figure 1:**
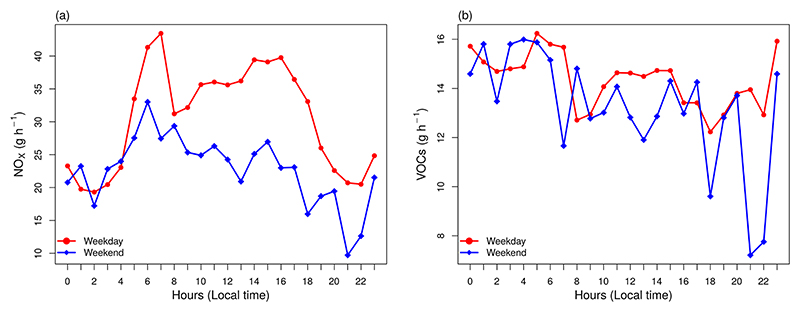
Mean emission from all street links from the Pinheiros neighborhood for (a) NO_*x*_ and (b) VOCs for a typical weekday and weekend.

Even when VEIN produces hourly emissions for a standard week (Fig. S2 in the Supplement), MUNICH only considers a standard day for weekdays and weekends. We choose Wednesday emission as a typical weekday and Saturday emission for the weekend. Figure 1 shows the mean diurnal profile of NO_*x*_ and VOCs emission fluxes from street links in the Pinheiros neighborhood.

#### 2.3.2 WRF simulation

**Figure 2:**
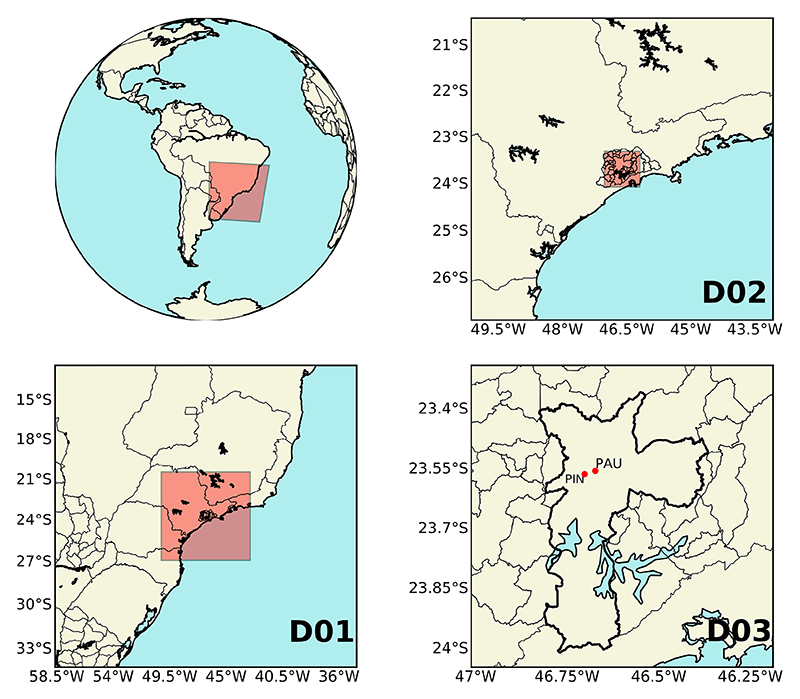
WRF simulation domains of 25 km (D01), 9 km (D02), and 1 km (D03) spatial resolution. D03 provides the meteorological information for MUNICH, the city of São Paulo is outlined in a thick black line, and the red dots show MUNICH domain locations.

Triple-nested domains are set up centered in SPMA. The mother domain has a spatial resolution of 25 km, the second 5 km, and the finest 1 km. The simulation at 1 km provides MUNICH with meteorological information. Initial and boundary conditions are retrieved from Historical Unidata Internet Data Distribution (IDD) Gridded Model Data (https://rda.ucar.edu/datasets/ds335.0/index.html, last access: 28 May 2020). Table 2 shows WRF configuration and Fig. 2 show the WRF domains.

Before using the WRF simulation outputs for MUNICH modeling, a model verification is performed. Model verification was carried out for the same period as MUNICH runs and for the finest domain output (D03). We used meteorological information from 16 air quality stations whose locations are shown in Fig. 4.

We also use benchmarks suggested by Emery et al. (2001), which were also used in Reboredo et al. (2015) and Pellegati Franco et al. (2019). However, Monk et al. (2019) explained that these benchmarks are suitable for domains in “simple” terrain; they also presented other sets of benchmarks for “complex” terrain, the latter being more suitable for SPMA. The results are detailed in Table 3. The temperature at 2 m (*T* 2) and relative humidity at 2 m (RH2) reach the simple terrain benchmarks, while wind speed and direction at 10 m (WS10 and WD10, respectively) are very close to them. When compared against complex terrain benchmarks, only the mean bias of WD10 is beyond the benchmark. Finally, *T* 2, RH2, and WS10 satisfy the good performance criteria of Keyser and Anthes (1977) and Pielke (2013). More details are shown in Tables S1 and S2 in the Supplement.

#### 2.3.3 Building height and street width

**Figure 3:**
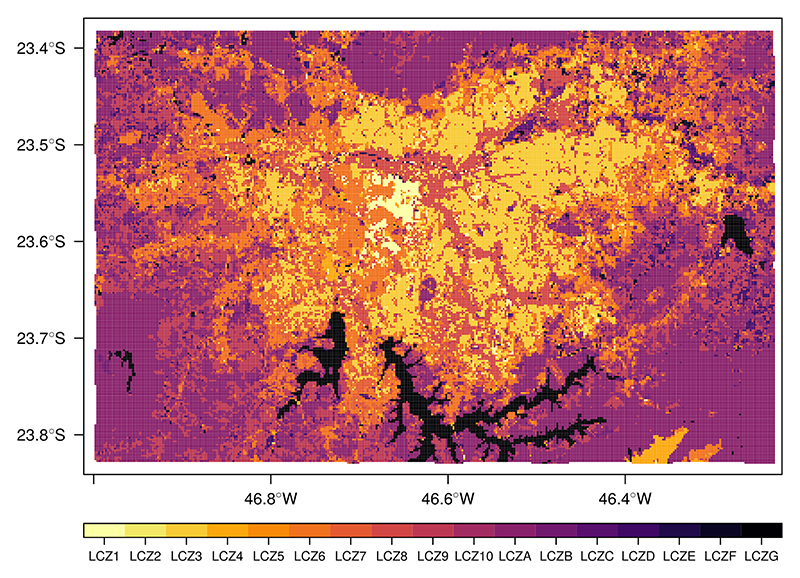
Local climate zones for SPMA.

Building height is retrieved from the World Urban Database and Access Portal Tools project (WUDAPT) for SPMA (Fig. 3). WUDAPT classifies urban areas into 17 local climate zones (LCZs). These LCZs are divided into build types, which are LCZs from 1 to 10, and land cover types, which go from A to G. Each of these LCZs presents different thermal, radiative, surface cover, and geometric properties. The building height is the height of roughness elements, which is the geometric average of building heights (Stewart and Oke, 2012). The WUDAPT file for SPMA is a raster with a spatial resolution of 120 m and was previously used in Pellegati Franco et al. (2019). Building height values for each LCZ are extracted from the URBPARM.TBL file from WRF-Chem simulations in Pellegati Franco et al. (2019) and assigned to the São Paulo WUDAPT raster file. The URBPRAM.TBL file contains the geomorphological and radiative parameters values for each LCZ based on Stewart et al. (2014).

The number of lanes is provided by the OpenStreetMap dataset, so the street width is calculated by using 3 m of lane width and by adding 1.9 m to each side of the street as side-walk width. Most OpenStreetMap streets do not include the number of lanes for this region; therefore, they are hole filled with the average by type of street.

#### 2.3.4 Background concentration

**Figure 4:**
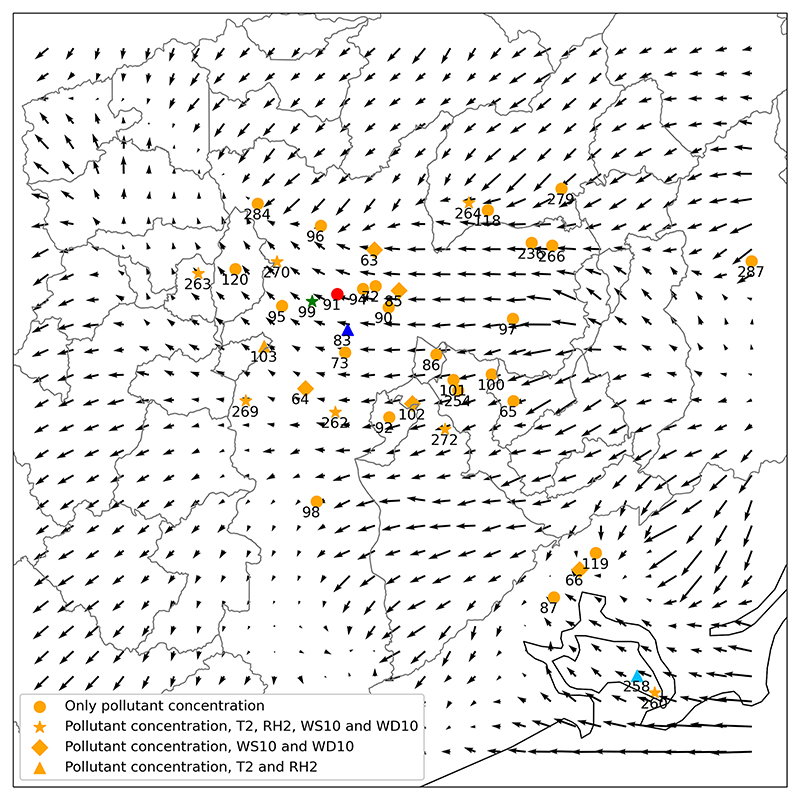
WRF average wind field for the simulation period with CETESB air quality stations (AQSs). The green star shows Pinheiros AQS (99), the red circle shows Cerqueira César AQS (91), and the blue triangle shows Ibirapuera AQS (83). Circles represent AQS that only measures pollutant concentrations; stars represent AQS that also measures *T* 2, RH2, WS10, and WD10; diamonds represents AQS that also measures WS10 and WD10; triangles represent AQS that also measures *T* 2 and RH2.

Vardoulakis et al. (2003) explained that the background concentration in street modeling is necessary to include the proportion of air pollutants that are not emitted inside the street. In the SinG model, background concentrations are the concentrations calculated by Polair3D, a mesoscale air quality model (Kim et al., 2018a). Wu et al. (2020) chose measurements from a station located very close to the study zone as the background concentration. Consequently, we consider the concentration outside the MUNICH domain as background concentration. With that in mind, by using the mean wind field from the WRF simulation for the study period, we select Ibirapuera air quality station (AQS) (83 shown in Fig. 4) measurements as background concentration, which, according to the wind field, advect pollutants to Pinheiros station (99) and Cerqueira César (83) as can be seen in Fig. 4. This assumption is only valid during daylight, when ozone concentrations are higher. As seen in Fig. S3 in the Supplement, during nighttime, wind presents a westerly direction. Measurements of O_3_, NO_2_, and NO in Ibirapuera AQS were used as background concentrations.

### 2.4 Measurements and statistical analysis

Meteorological and air pollutant measurements are retrieved from the CETESB air quality network. To evaluate WRF simulation in the finest domains, observations from 16 AQSs are used. Background concentration comes from the Ibirapuera AQS. The Pinheiros AQS is used to evaluate MUNICH performance in the Pinheiros neighborhood, while Cerqueira César is used to evaluate Paulista Avenue. To evaluate model performance, we follow the recommendations from Emery et al. (2017). We also use the evaluation statistics from Hanna and Chang (2012): fractional bias (FB), normalized mean square error (NMSE), fraction of predictions within a factor of 2 (FAC2), and normalized absolute difference (NAD). The acceptance criteria for urban zones are |FB| < = 0.67, NMSE < = 6, FAC2 > = 0.3, and NAD < = 0.5. We expand the statistical analysis to the background concentration to see the difference against observations and to assess the influence of background concentration in MUNICH simulations.

### 2.5 Model set up

**Figure 5:**
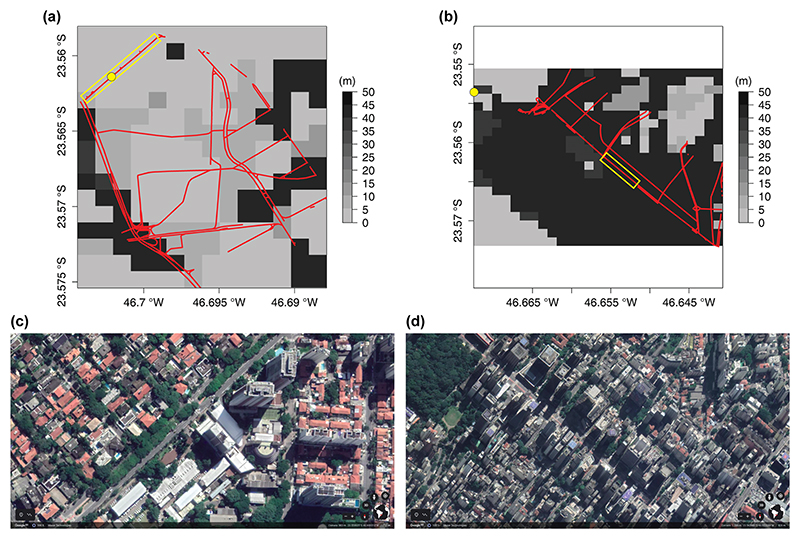
Pinheiros neighborhood **(a)** and Paulista Avenue **(b)** MUNICH domains and building height; the red lines are the streets considered in VEIN; the yellow dot shows Pinheiros AQS and Cerqueira César (AQS). Yellow squares highlight the selected urban canyon for comparison against observations. At the bottom, satellite photos are shown of those urban canyons (source: © Google Maps 2019).

We use MUNICH to simulate two urban areas inside SPMA: the first domain is the Pinheiros neighborhood and the second one is Paulista Avenue. VEIN produces emissions for all the street links in SPMA. This information can be filtered by the neighborhood name of the street links. We subset that information for the Pinheiros neighborhood (Fig. 5a) and for the neighborhoods that contain the Paulista Avenue urban canyon (Fig. 5b). In MUNICH, NO emissions are estimated from NO_*x*_ and NO_2_ emissions.

Figure 5 shows MUNICH domain for the Pinheiros neighborhood and Paulista Avenue. The yellow dot represents the location of the air quality stations. The red lines are the street links used by VEIN to calculate the emissions, and the yellow rectangle is the urban canyon selected for comparison against observations.

There are 677 street links for Pinheiros and 535 for Paulista Avenue. In total, nine points of WRF simulation cover the Pinheiros domains, while 12 WRF points represent Paulista Avenue domains. From WUDAPT, we can see that inside Pinheiros there is a variety of buildings with different heights. The Pinheiros AQS is located in an urban canyon that has a mean building height of 5 m (LCZ 6 – open low rise). On the other hand, the Paulista Avenue domain is more uniform, presenting urban canyons with a mean building height of 45 m (LCZ1 – compact high rise).

## 3 Results

Here, we present the O_3_ and NO_*x*_ simulations with MUNICH for a week in October 2014. We first calibrated the input emissions by studying the Pinheiros neighborhood to later simulate NO_*x*_ inside the Paulista Avenue urban canyon.

### 3.1 Control case for the Pinheiros neighborhood

**Figure 6:**
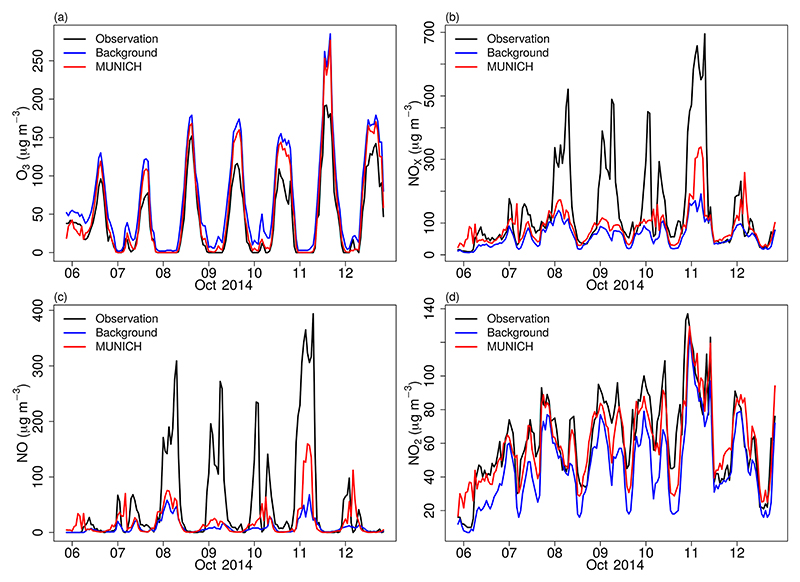
Comparison of MUNICH results against background and observation concentrations of (a) O_3_, (b) NO_*x*_, (c) NO, and (d) NO_2_ for the Pinheiros urban canyon from the control case.

Figure 6 shows the results of MUNICH simulation using the original emissions calculated by VEIN for SPMA. MUNICH simulations are very close to background concentrations, which leads to an overprediction of O_3_ and underpredicted NO and NO_*x*_ concentrations. This is produced by a dependence of MUNICH on background concentration and by emission underestimation. The emission underestimation is caused by emission factors calculated based on average measurements of emissions certification tests and because emission factors derived from a dynamometer, and cycle measurements do not represent real-drive emissions (Ropkins et al., 2009). It is also probable that the number of vehicles could have been underestimated inside the urban canyon. The underestimation of NO_*x*_ is caused by the underestimation of NO concentrations. NO_2_ concentration magnitude is well represented by MUNICH.

**Figure 7:**
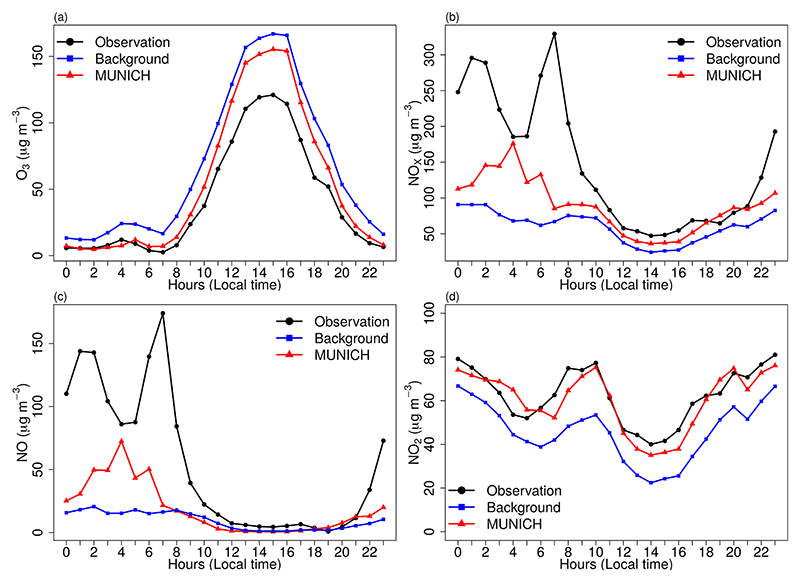
Diurnal profile of MUNICH results, background, and concentrations of (a) O_3_, (b) NO_*x*_, (c) NO, and (d) NO_2_ for the Pinheiros urban canyon from the control case.

The diurnal variations of MUNICH simulation, observation, and background concentrations are shown in Fig. 7. MUNICH coherently simulated the temporal variation of O_3_ and NO_2_ concentrations inside the urban canyon. For NO and NO_*x*_, the temporal variation during the day and until mid-night is well simulated, while the morning peak at 06:00 LT is underestimated. After midnight, a higher concentration of NO_*x*_ occurs with the increase of heavy-duty vehicles at night that mainly run on diesel. In Pinheiros urban canyons, there is a predominant flow of light-duty vehicles, even though it is registered high NO_*x*_ concentrations that it is transported from the highway. The mean differences between MUNICH simulation and background concentration for O_3_, NO_*x*_, NO, and NO_2_ are −13.10, 28.61, 9.25, and 14.43 μg m^−3^, respectively.

### 3.2 Emission adjustment

**Figure 8:**
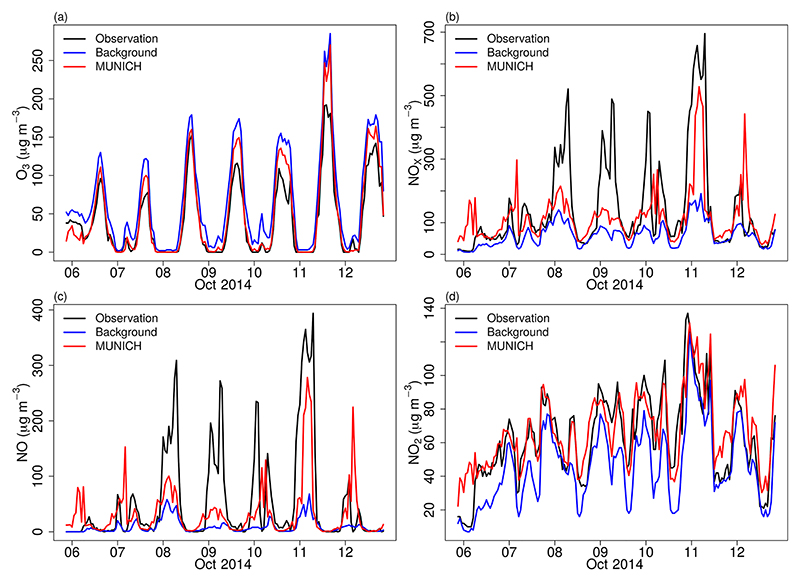
Comparison of MUNICH results against background and observation concentrations of (a) O_3_, (b) NO_*x*_, (c) NO, and (d) NO_2_ for the Pinheiros urban canyon from the MUNICH-Emiss simulation.

We ran different scenarios with increased NO_*x*_ and VOC emission from VEIN. The best results were produced when we doubled the NO_*x*_ and VOC emissions; this scenario is called MUNICH-Emiss. With this adjustment, we achieved an overall improvement of MUNICH simulations. Figure 8 shows the new comparison between the model, background concentration, and observations. O_3_ is still overpredicted, which is caused by the higher value of O_3_ background concentration together with a low NO background concentration; nevertheless, the simulated O_3_ concentration during nighttime is well represented and daily peaks values are closer to observations.

NO_*x*_ and NO simulations are still underpredicted, but NO_2_ is of the same magnitude as observations. NO_*x*_ underprediction is still mainly attributed to the underprediction of NO, especially during 8, 9, and 10 October when high observational values of NO were recorded. NO underestimation is explained by the lower NO background concentration, the underestimation of emissions, and the use of a single-day emission profile to represent all weekdays. Wind speed over-estimation also affects this underestimation as it enhances dispersion. However, MUNICH can better represent the observed high concentration during Saturday, October 11, as MUNICH uses the same emission profile for the weekend and weekdays; this high simulated NO concentration resulted from the influence of meteorology.

**Figure 9:**
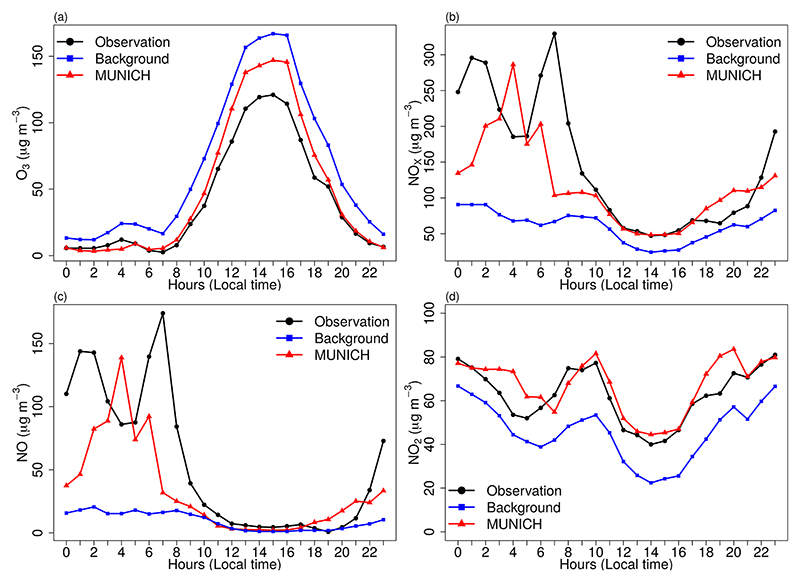
Diurnal profile of MUNICH results, background, and concentration for (a) O_3_, (b) NO_*x*_, (c) NO, and (d) NO_2_ for the Pinheiros urban canyon from the MUNICH-Emiss simulation.

Figure 9 shows the diurnal profiles for this simulation. The new MUNICH-Emiss profiles are closer to observed concentration profiles, with a better representation of the peak concentration magnitude of NO_*x*_, NO, and NO_2_. The mean differences over the simulation period between simulated and the background concentrations for O_3_, NO_*x*_, NO, and NO_2_ are −17.85, −57.26, 23.60, and 21.07 μg m^−3^, respectively, showing bigger differences than the control case previous scenario and the influence of the reaction with NO emissions.

Table 4 summarizes the performance statistics for each scenario and background. The performance statistics from the MUNICH-Emiss case show lower values of MB, NMGE, and RMSE for all pollutants, except NO_2_ which presents a slight increase in these indicators. They also show high values of *R* (≥ 0.7) for each pollutant in every case, which indicates that the temporal variations of emission and background concentration are in the same phase as the observations. In general, in both MUNICH simulations, NO_2_ and O_3_ are better simulated. The MUNICH-Emiss case performs better and also achieves the recommendations of Hanna and Chang (2012) for O_3_, NO_2_ NO, and NO_*x*_, whereas the MUNICH control case did not reach these recommendations for NO.

**Figure 10:**
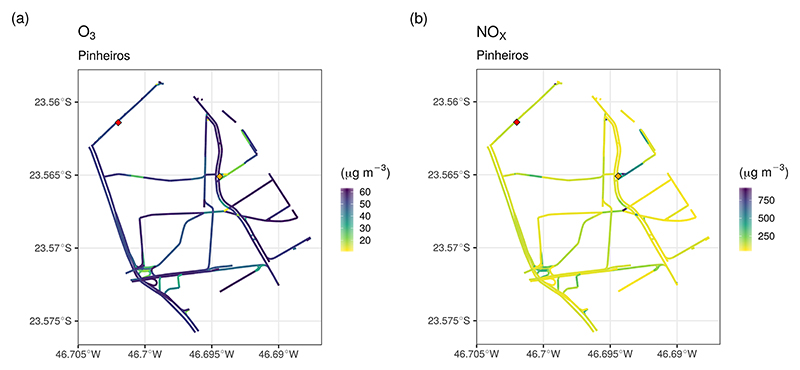
Hourly mean simulated concentration of (a) O_3_ and (b) NO_*x*_ for the Pinheiros neighborhood. The red diamond denotes the location of the Pinheiros AQS and the orange diamond denotes traffic light location.

Figure 10 shows the mean hourly concentration of O_3_ and NO_*x*_ in the Pinheiros neighborhood; the red diamond points to the location of Pinheiros air quality station. Because the VEIN model can distribute spatially the emissions, there is a variation of concentrations in different street links. For example, the orange diamond shows the location of a traffic light, where traffic jams occur, causing lower O_3_ concentrations from higher NO_*x*_ emissions.

We also perform an additional sensitivity simulation by running the MUNICH scenario using the background concentrations from the Santos AQS (light blue triangle in Fig. 4). Compared to the Ibirapuera AQS site, measured O_3_ and NO_2_ concentrations are lower, and those of NO concentrations are higher at the Santos AQS. This results in O_3_ and NO_2_ underprediction and a better simulation of NO concentration magnitude; however, all evaluated pollutants present lower *R* values and higher NMGE values than MUNICH-Emiss scenario with Ibirapuera AQS as the background concentration. Simulated NO_2_ and O_3_ follow background concentrations, which indicates that the MUNICH simulations have a strong dependence on the background concentration (see Figs. S4 and S5 in the Supplement).

Lastly, a sensitivity simulation was performed with an only increase of NO_*x*_ emissions by four and remaining VOC original emission using Ibirapuera background concentration. This results in a better O_3_ representation but unrealistic NO_*x*_, NO, and NO_2_ concentration (see Figs. S6 and S7 in the Supplement). As SPMA has a VOC-limited regime (Andrade et al., 2017), the increment of NO_*x*_ emission will lead to a reduction of O_3_ concentration. Many studies have shown that São Paulo’s atmosphere is VOC limited (Schuch et al., 2020) due to the high NO_*x*_ emission by the heavy-duty vehicles that are under old emissions regulations. The new regulations for diesel engine emissions were established recently and are being implemented according to the recycling of the fleet, which is 20 years of use for diesel trucks (CETESB, 2019).

### 3.3 Application for Paulista Avenue

**Figure 11:**
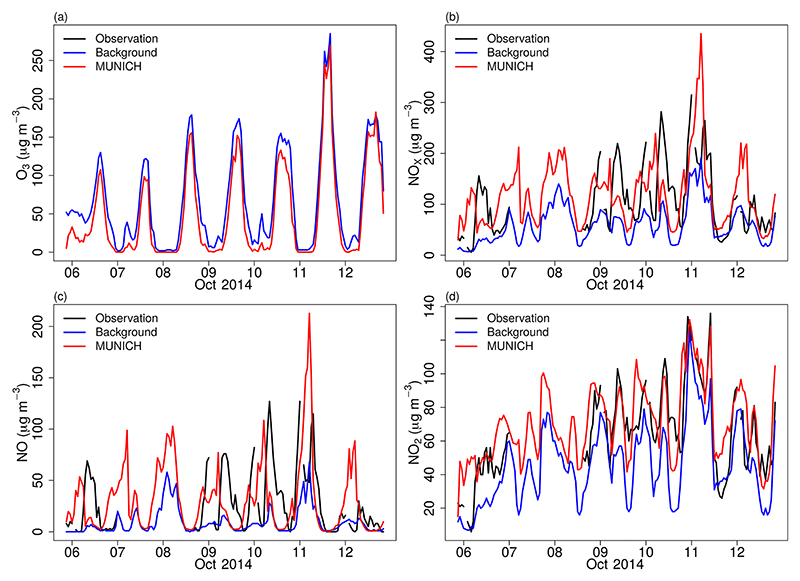
Comparison of MUNICH results against background and observation concentration for (a) O_3_, (b) NO_*x*_, (c) NO, and (d) NO_2_ for the Paulista Avenue urban canyon. Note that O_3_ observations were not available for the Paulista Avenue domain.

**Figure 12:**
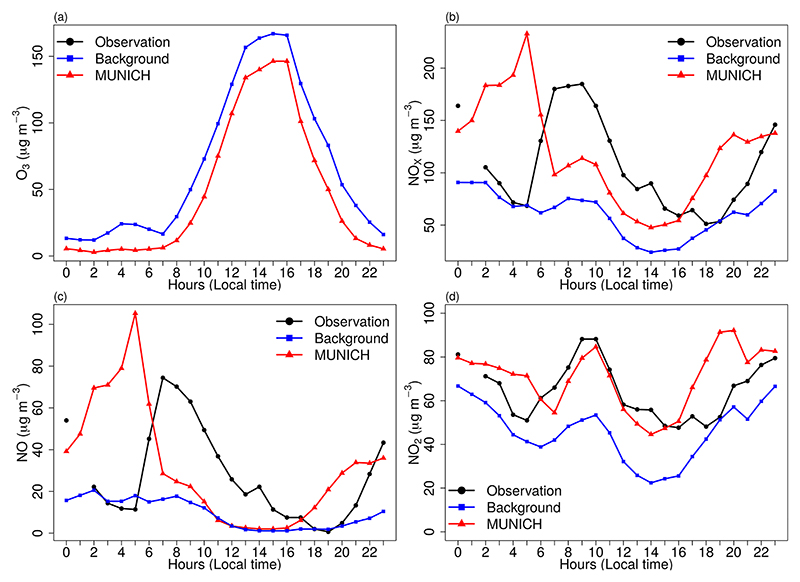
Diurnal profile of MUNICH results, background, and concentration for (a) O_3_, (b) NO_*x*_, (c) NO, and (d) NO_2_ for Paulista Avenue. Note that O_3_ observations were not available for the Paulista Avenue domain.

The MUNICH simulation is performed with calibrated emissions for a domain that contains a well-defined urban canyon: Paulista Avenue. The simulation shows a better representation of NO_*x*_, NO, and NO_2_ temporal variations and a good representation of concentration magnitude (Fig. 11). Although the MB indicates an overprediction of NO_*x*_, NO, and NO_2_ (Table 5), Fig. 12 shows that this is caused by an overprediction of these pollutants during night hours, linked to a mismatch of emissions. As in the Pinheiros domain, MUNICH did not capture the two peaks of NO and NO_*x*_ during nighttime. This is caused by WRF limitation in representing planetary boundary layer height during nighttime (Hu et al., 2012; McNider and Pour-Biazar, 2020). Also, as shown in Fig. 1a, the NO_*x*_ emission profile during weekdays presents two peaks during daylight at 07:00 and 16:00 LT (local time) and a smaller emission peak around 23:00 h; it is probable that this nighttime peak was underestimated.

Statistics in Table 5 show an improvement in representing concentration magnitudes of NO_*x*_, NO, and NO_2_ with mean simulated concentrations close to observations and very low values of MB, NMB, and RMSE. In this case, *R* values are lower than those in the Pinheiros case but still higher than 0.4 for NO_*x*_ and NO_2_, confirming that there is a mismatch of simulated concentrations, which is clearer in the MUNICH NO_*x*_ and NO peak happening before observation. The MUNICH-Emiss simulations achieve Hanna and Chang (2012) performance criteria for NO_*x*_ and NO_2_. NO_2_ is the best simulated species.

## 4 Discussion and conclusions

Simulating air pollutants inside urban street canyons is a challenging task. It is even more difficult in cities as heterogeneous as São Paulo, where its urban structure is not always textbook defined. The limited number of air quality stations located inside or near urban canyons, together with the lack of information from detailed emission inventories and urban morphology data, hinders accurate air quality modeling and consequently air quality management.

In this paper, we attempt to fill in this gap by using the MUNICH street-network model together with the VEIN vehicular emissions model. The latter provides temporal and spatially detailed emission fluxes inside the main streets and coordinates and width of the streets (i.e., the street network). The urban morphology is completed by extracting the building height from the WUDAPT database for the São Paulo metropolitan area. The advantages of using MUNICH are that, besides solving pollutant dispersion, it also solves photochemistry reactions and is an operational model that solves pollutant concentration at neighborhood scale considering street intersections.

Results showed that MUNICH simulations that used adjusted emissions can better represent the temporal variation of O_3_, NO_*x*_, NO, and NO_2_ concentrations inside urban canyons. Nevertheless, the results are highly dependent on background concentrations and emission fluxes. This background concentration dependence is stronger in secondary pollutants such as O_3_, and primary pollutants are more determined by emission fluxes. The reason for the significant contribution of background concentration is that MUNICH is based in SIRANE, and SIRANE also presents a significant contribution from background concentration (Soulhac et al., 2012).

The main cause of O_3_ overprediction in our simulation for both tested urban zones is the high value of background O_3_ concentration measured in the Ibirapuera AQS. In the Pinheiros neighborhood, the underprediction of NO_*x*_ concentration is caused by the underprediction of NO concentration in Pinheiros during the second half of the week. This underestimation is caused by the lower NO background concentration together with an emission underestimation. The concentration magnitudes in Paulista Avenue are well represented but there was a mismatch with observed concentration. MUNICH-Emiss scenario fulfills the performance criteria. O_3_ concentration simulated in Pinheiros and Paulista Avenue is lower than background concentrations; these same results are reported by Wu et al. (2019). As noted in Krecl et al. (2016), this behavior is caused by the high NO_*x*_ emissions inside the street urban canyons, which rapidly deplete the formed O_3_ and the one from the rooftop (i.e., background concentration).

As the main source of surface NO and NO_2_ emissions in São Paulo are vehicles, it is necessary to go deeper into the reasons why the MUNICH-Emiss scenario performs better. The increase of the emissions is necessary because the emissions factors are the average of emission certification tests (CETESB, 2015). It has been shown that emission factors derived from dynamometer and cycle measurements do not represent real-drive emissions (Ropkins et al., 2009). São Paulo does not have an inspection and maintenance (I&M) program; therefore, there may exist a fraction of the fleet which are high emitters and do not meet the emission standards; more details can be found in Ibarra-Espinosa et al. (2020a). Furthermore, the comparison of traffic flow between GPS and TDM data for the Pinheiros area showed that TDM traffic flows are 2.22 times higher than GPS. Hence, more representative traffic flows would also improve the emissions compilation. As a conclusion, it is important to develop new and more representative vehicular traffic flow and emission factors for Brazil.

With calibrated emissions (i.e., MUNICH-Emiss scenario), the good performance of MUNICH in representing NO_2_ concentrations in both neighborhoods and NO and NO_*x*_ in Paulista Avenue urban canyon suggests that the VEIN model distributes emissions spatially and temporally efficiently, which proves its potential to be used in other cities. VEIN is being continuously developed and currently offers some utilities to format emissions to the MUNICH model. On the other hand, now Google Earth allows new features such as 3-D view that together with in situ measurements can improve WUDAPT building height estimates. These new features can be used to improve MUNICH input data and therefore the model simulation results. Further, a better estimation of background concentrations from photochemical grid models can potentially improve the model performance.

The results obtained show the promising capability of MUNICH to represent the concentrations of pollutants emitted by the fleet close to the streets. As MUNICH uses the CB05 gas-phase mechanism, it can also simulate VOCs inside the urban canyon. Measurements of VOCs inside urban canyons are therefore necessary to validate the model in the future. An accurate prediction of street-scale air pollutant concentrations will enable the future assessment of the impacts on human health due to their exposure to air pollutants emitted by the vehicles.

## Supplementary Material

Appendix

## Figures and Tables

**Table 1:** Summarized MUNICH input data.

Input data	Source
Meteorological input	WRF 3.7.1 simulation centered in SPMA (DX = 1 km)
Street links coordinates and with lanes number	VEIN emission model (Ibarra-Espinosa et al., 2018)
Street links emissions	VEIN emission model (Ibarra-Espinosa et al., 2018)
Building height	World Urban Database and Access Portal Tools project (WUDAPT) database for SPMA (http://www.wudapt.org/, last access: 28 May 2020)
Background concentration	O_3_, NO, and NO_2_ from the Ibirapuera air quality station (AQS)
VOC speciation	Ethanol, formaldehyde and acetaldehyde from WRF-Chem emission file from Andrade et al. (2015); other species are based on concentration shown in Dominutti et al. (2016)

**Table 2:** WRF simulation configuration.

Attribute	Configuration
WRF version	3.7.1
Domains spatial resolution	DX = 25, 5, and 1 km
Simulation period	3 to 13 October 2014 (first 3 d are a spin-up period and not analyzed)
Meteorological IC/BC	Historical Unidata IDD gridded model data (ds335.0)
Longwave radiation	RRTMG scheme (Iacono et al., 2008)
Shortwave radiation	RRTMG scheme (Iacono et al., 2008)
Planetary boundary layer (PBL)	Yonsei University (YSU) scheme (Hong et al., 2006)
Surface layer	Noah model (Tewari et al., 2004)
Cumulus cloud	Multi-scale Kain–Fritsch scheme (Zheng et al., 2016)
Cloud microphysics	Morrison double-moment scheme (Morrison et al., 2009)

**Table 3:** WRF statistical model verification of simulation quality.

Parameter	Benchmark simple terrain	Benchmark complex terrain	Value from the WRF simulation
Temperature at 2 m	MB* < ± 0.5 K MAGE < 2.0 K IOA ≥ 0.8	MB < ± 1.0K MAGE < 3.0 K	0.27 K 1.59 K 0.83 K
Relative humidity at 2 m	MB < ± 10.0 % MAGE < 20 % IOA > 0.6		−5.02% 9.79 % 0.74
Wind speed at 10 m	MB < ± 0.5 ms^−1^ RMSE ≤ 2 ms^−1^	MB < ± 1.5 ms^−1^ RMSE ≤ 2.5 ms^−1^	0.79 ms^−1^ 1.59 ms^−1^
Wind direction at 10 m	MB < ± 10.0° MAGE < 30°	MB < ± 10.0° MAGE < 55°	**–16.23**° 5°

* MB: mean bias, MAGE: mean absolute gross error, IOA: index of agreement, and RMSE: root mean square error. Results outside the benchmark are highlighted in bold.

**Table 4:** Statistical indicators for O_3_, NO_*x*_, NO, and NO_2_ for comparison between background concentration, the MUNICH simulation, and MUNICH-Emiss against observations from Pinheiros AQS.

		$\bar{M}$*	$\bar{O}$	*σ*_M_	*σ*_O_	MB	NMB	NMGE	RMSE	*R*	|FB|	NMSE	FAC2	NAD
O_3_	Background	67.6	41.5	63.2	47.5	26.1	0.6	0.6	32.4	0.98	**0.5**	**0.4**	**0.5**	**0.2**
	MUNICH	54.5	41.5	62.1	47.5	13.0	0.3	0.3	22.2	0.98	**0.3**	**0.2**	**0.6**	**0.1**
	MUNICH-Emiss	49.7	41.5	59.5	47.5	8.2	0.2	0.3	18.0	0.98	**0.2**	**0.2**	**0.6**	**0.1**
NO_*x*_	Background	60.3	146.4	37.3	150.3	−86.0	−0.6	0.6	149.6	0.79	0.8	**2.5**	**0.5**	**0.4**
	MUNICH	88.9	146.4	57.4	150.3	−57.4	−0.4	0.5	128.5	0.70	**0.5**	**1.3**	**0.7**	**0.2**
	MUNICH-Emiss	117.6	146.4	85.6	150.3	−28.8	−0.2	0.5	120.0	0.60	**0.2**	**0.8**	**0.7**	**0.1**
NO	Background	9.5	54.6	12.7	88.9	−45.1	−0.8	0.8	91.5	0.75	1.4	16.2	**0.3**	0.7
	MUNICH	18.7	54.6	28.7	88.9	−35.9	−0.7	0.8	80.7	0.70	1.0	6.4	0.1	**0.5**
	MUNICH-Emiss	33.1	54.6	48.5	88.9	−21.5	−0.4	0.8	74.5	0.60	**0.5**	**3.1**	**0.3**	**0.2**
NO_2_	Background	45.8	62.7	23.4	25.9	−16.8	−0.3	0.3	21.2	0.87	**0.3**	**0.2**	**0.9**	**0.2**
	MUNICH	60.3	62.7	22.8	25.9	−2.4	0.0	0.2	13.3	0.90	**0.0**	**0.0**	**1.0**	**0.0**
	MUNICH-Emiss	66.9	62.7	22.0	25.9	4.2	0.10	0.2	14.8	0.80	**0.1**	**0.1**	**0.9**	**0.0**

$\bar{M}$ – model value mean (μg m^−3^), $\bar{O}$ – Observation mean (μg m^−3^), *σ*_M_ – model standard deviation (μg m^−3^), *σ*_O_ – observation standard deviation (μg m^−3^), MB – mean bias (μg m^−3^), NMB – normalized mean bias, NMGE – normalized mean gross error, RMSE – root mean square error (μg m^−3^), *R* – correlation coefficient, FB – fractional mean bias, NMSE – normalized mean square error, FAC2 – fraction of predictions within a factor of 2, and NAD – normalized absolute difference. Values in bold satisfied Hanna and Chang (2012) acceptance criteria.

**Table 5:** Statistical indicators for O_3_, NO_*x*_, NO, and NO_2_ for comparison between background concentration and MUNICH-Emiss against observations from the Cerqueira César AQS.

		$\bar{M}$ *	$\bar{O}$	*σ*_M_	*σ*_O_	MB	NMB	NMGE	RMSE	*R*	|FB|	NMSE	FAC2	NAD
NO_*x*_	Background	56.8	105.8	36.6	66.8	−49.0	−0.5	0.5	68.9	0.7	**0.6**	0.8	**0.6**	**0.3**
	MUNICH-Emiss	114.8	105.8	68.4	66.8	9.0	0.1	0.6	74.2	0.4	**0.1**	**0.5**	**0.7**	**0.0**
NO	Background	7.3	26.9	10.3	30.7	−19.6	−0.7	0.8	32.5	0.6	1.1	**5.3**	0.2	0.6
	MUNICH-Emiss	28.0	26.9	35.2	30.7	1.1	0.0	1.1	40.8	0.2	**0.0**	2.2	0.2	**0.0**
NO_2_	Background	45.5	64.6	24.3	26.5	−19.0	−0.3	0.3	24.2	0.8	**0.3**	**0.2**	**0.8**	**0.2**
	MUNICH-Emiss	71.9	64.6	23.9	26.5	7.4	0.10	0.2	19.1	0.8	**0.1**	**0.1**	**0.9**	**0.1**

* $\bar{M}$ – model value mean (μg m^−3^), $\bar{O}$ – observation mean (μg m^−3^), *σ*_M_ – model standard deviation (μg m^−3^), *σ*_O_ – observation standard deviation (μg m^−3^), MB – mean bias (μg m^−3^), NMB – normalized mean bias, NMGE – normalized mean gross error, RMSE – root mean square error (μg m^−3^), *R* – correlation coefficient, FB – fractional mean bias, NMSE – normalized mean square error, FAC2 – fraction of predictions within a factor of 2, and NAD – normalized absolute difference. Values in bold satisfied Hanna and Chang (2012) acceptance criteria.

## Data Availability

MUNICH input and output data and scripts to generate the figures and calculations are available on GitHub (https://github.com/quishqa/MUNICH_VEIN_SP, last access: 28 May 2021) and Zenodo (https://doi.org/10.5281/zenodo.4168056, Gavidia Calderón, 2020). MUNICH (v1.0) is available on http://cerea.enpc.fr/munich/index.html and Zenodo (https://doi.org/10.5281/zenodo.4168985, Kim et al., 2018b). VEIN can be installed from CRAN, and it is also available on Zenodo (https://doi.org/10.5281/zenodo.3714187, Ibarra-Espinosa et al., 2020b). Additional information and help are available by contacting the authors.
