# Supplementary material for: Simulation of O3 and NOx in São Paulo street urban canyons with VEIN (v0.2.2) and MUNICH (v1.0)
Source: Geosci Model Dev. Author manuscript; Available in PMC 2024 May 29. (PMC7615997; doi:10.5194/gmd-14-3251-2021)
Supplement: Appendix [file EMS196215-supplement-Appendix.pdf]

Appendix A: Statistical indicators

Table A1. Statistical indicator definition.

Statistical indicator	Definition	Reference
Fraction of prediction within a factor of 2 (FAC2)	$\mathrm{FAC2} = 0.5 \leq \frac{M_i}{O_i} \leq 2.0$	Emery et al. (2017)
Mean bias (MB)	$\mathrm{MB} = \frac{1}{N}\sum_{i=1}^{N}(M_i - O_i)$	Emery et al. (2017)
Mean absolute gross error (MAGE)	$\mathrm{MAGE} = \frac{1}{N}\sum_{i=1}^{N}\lvert M_i - O_i\rvert$	Emery et al. (2017)
Normalized mean bias (NMB)	$\mathrm{NMB} = \frac{\sum_{i=1}^{N}(M_i - O_i)}{\sum_{i=1}^{N} O_i}$	Emery et al. (2017)
Normalized mean error (NME)	$\mathrm{NME} = \frac{\sum_{i=1}^{N}\lvert M_i - O_i\rvert}{\sum_{i=1}^{N} O_i}$	Emery et al. (2017)
Root mean square error (RMSE)	$\mathrm{RMSE} = \sqrt{\frac{1}{N}\sum_{i=1}^{N}(M_i - O_i)^2}$	Emery et al. (2017)
Correlation coefficient (R)	$R = \frac{1}{(N-1)}\sum_{i=1}^{N}\left(\frac{M_i - \overline{M}}{\sigma_{\mathrm{M}}}\right)\left(\frac{O_i - \overline{O}}{\sigma_{\mathrm{O}}}\right)$	Emery et al. (2017)
Fractional mean bias (FB)	$\mathrm{FB} = 2.0\frac{\overline{O_i - M_i}}{\overline{O} + \overline{M}}$	Hanna and Chang (2012)
Normalized mean square error (NMSE)	$\mathrm{NMSE} = \frac{\overline{(O_i - M_i)^2}}{\overline{O} \times \overline{M}}$	Hanna and Chang (2012)
Normalized absolute difference (NAD)	$\mathrm{NAD} = \frac{\overline{\lvert O_i - M_i\rvert}}{\overline{O} + \overline{M}}$	Hanna and Chang (2012)

Data availability. MUNICH input and output data and scripts to generate the figures and calculations are available on GitHub (https://github.com/quishqa/MUNICH_VEIN_SP, last access: 28 May 2021) and Zenodo (https://doi.org/10.5281/zenodo.4168056, Gavidia Calderón, 2020). MUNICH (v1.0) is available on http://cerea.enpc.fr/munich/index.html and Zenodo (https://doi.org/10.5281/zenodo.4168985, Kim et al., 2018b). VEIN can be installed from CRAN, and it is also available on Zenodo (https://doi.org/10.5281/zenodo.3714187, Ibarra-Espinosa et al., 2020b). Additional information and help are available by contacting the authors.

Supplement. The supplement related to this article is available online at: https://doi.org/10.5194/gmd-14-3251-2021-supplement.
